# Supplementary material for: Association between oral microbiome and seven types of cancers in East Asian population: a two-sample Mendelian randomization analysis
Source: Front Mol Biosci. 2023 Nov 21;10:1327893. doi: 10.3389/fmolb.2023.1327893 (PMC10702768; doi:10.3389/fmolb.2023.1327893)
Supplement: Supplementary file 1 [file DataSheet1.ZIP › Supplementary Materials/MR plots for tongue/tongue═╝/Ovarian cancer/pheno.2184_to_ovarian cancer_forest.pdf]

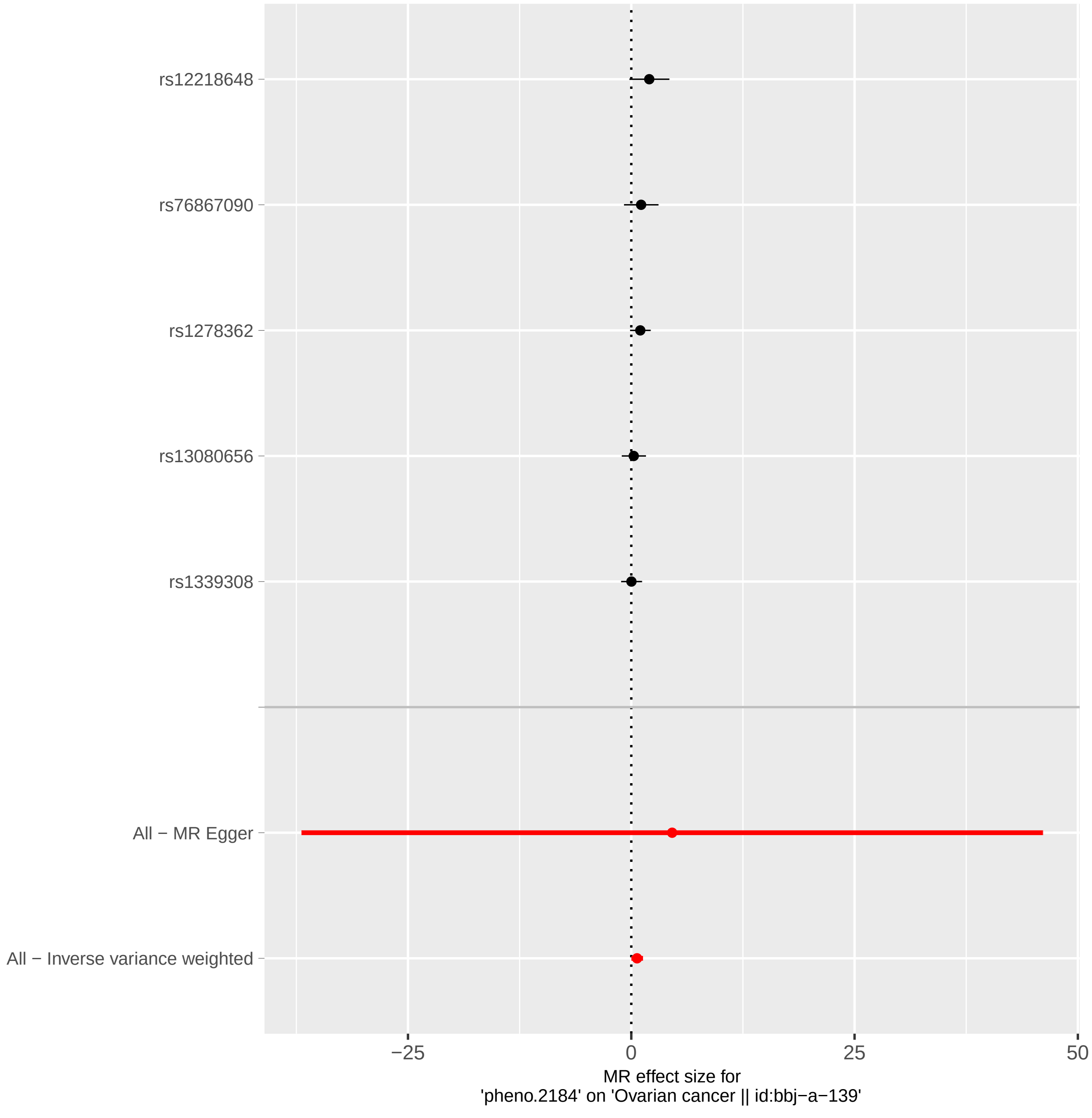
rs12218648
rs76867090
rs1278362
rs13080656
rs1339308
All − MR Egger
All − Inverse variance weighted
−25
0
25
50
MR effect size for
'pheno.2184' on 'Ovarian cancer || id:bbj−a−139'
